# Congenital Scoliosis in Smith–Magenis Syndrome: A Case Report and Review of the Literature

**DOI:** 10.1097/MD.0000000000000705

**Published:** 2015-05-01

**Authors:** Zheng Li, Jianxiong Shen, Jinqian Liang, Lin Sheng

**Affiliations:** From the Department of Orthopaedic Surgery, Peking Union Medical College Hospital, Chinese Academy of Medical Sciences and Peking Union Medical College, Beijing, China.

## Abstract

The Smith–Magenis syndrome (SMS) is a complex and rare congenital condition that is characterized by minor craniofacial anomalies, short stature, sleep disturbances, behavioral, and neurocognitive abnormalities, as well as variable multisystemic manifestations. Little is reported about spinal deformity associated with this syndrome.

This study is to present a case of scoliosis occurring in the setting of SMS and explore the possible mechanisms between the 2 diseases.

The patient is a 13-year-old Chinese female with congenital scoliosis and Tetralogy of Fallot, mental retardation, obstructive sleep apnea, hypertrophy of tonsil, conductive hearing loss, and agenesis of the epiglottis. An interphase fluorescent in situ hybridization at chromosome 17p11.2 revealed a heterozygous deletion, confirming a molecular diagnosis of SMS. She underwent a posterior correction at thoracic 1-lumbar 1 (T1-L1) levels, using the Moss-SI spinal system. At 6-month follow-up, the patient was clinically pain free and well balanced. Plain radiographs showed solid spine fusion with no loss of correction.

Congenital cardiac disease, immunodeficiency, and severe behavioral problems can affect the surgical outcome following spine fusion and need to be taken into consideration for the surgeon and anesthesiologist. Scoliosis is not uncommon among patients with SMS, and there is a potential association between congenital scoliosis and SMS. The potential mechanisms in the pathogenesis of congenital scoliosis of SMS included retinoic acid-induced 1 (RAI1) microdeletion and *RAI1* gene point mutation.

## INTRODUCTION

Smith–Magenis syndrome (SMS) is a multisystem disorder characterized minor craniofacial anomalies, sleep disturbances, short stature, behavioral, and neurocognitive abnormalities, as well as variable multisystemic manifestations.^1–3^ The prevalence of SMS is estimated at 1:15,000 to 1:25,000 births.^3,4^ SMS is a complex, contiguous gene syndrome associated with an interstitial microdeletion at the 17p11.2 chromosome region that includes the retinoic acid-induced 1 (*RAI1*) gene (around 90% of cases) or by *RAI1* gene point mutation (10% of cases of SMS).^5,6^ The gene functions of *RAI1* are linked to regulation of several genes that control specific pathways of various biological processes.^7^ This syndrome was first described by Smith et al in 1982, in 2 patients with cleft palate and congenital heart disease.^8^ There have been a number of reports describing various skeletal features in this syndrome, including brachydactyly, short stature, cleft lip/palate, persistent fetal finger pads, and polydactyly.^8–10^ However, there are limited reports regarding the association with SMS and congenital scoliosis. We here present a case of SMS in a 13-year-old girl with an unusual presentation, congenital scoliosis.

## CONSENT

Written informed consent was obtained from the patient's parents on behalf of the child for publication of this case report and any accompanying images. A copy of the written consent is available for review by the editor of this journal.

## CASE REPORT

**FIGURE 1:**
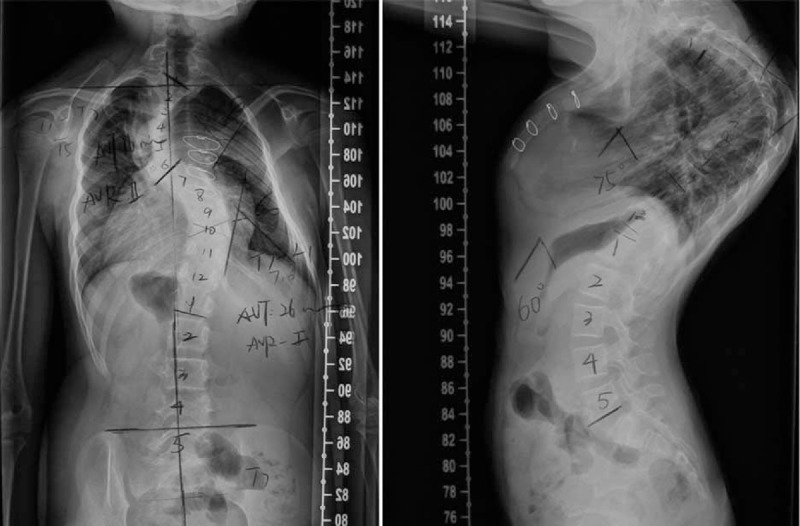
Standing anteroposterior and lateral radiographs of the preoperation.

We present the case of a 13-year-old girl who was admitted for the correction of her scoliosis. Her plain radiographs of the spine showed a thoracic scoliosis with a Cobb angle of 74° (Figure 1), suggesting the need for surgical correction.

Her past medical history was remarkable in that the girl underwent total correction of Tetralogy of Fallot at the age of 8 year in another hospital. On clinical examination, the patient was found to be mentally retarded. Some sleep problems were reported by the parents, including snoring, shortness of breath, and gasping to awake. So, she was referred to the Department of Otorhinolaryngology in our hospital and found that she had obstructive sleep apnea, hypertrophy of tonsil, conductive hearing loss, and agenesis of epiglottis. An interphase fluorescent in situ hybridization at chromosome 17p11.2 revealed a heterozygous deletion, confirming a molecular diagnosis of SMS.

**FIGURE 2:**
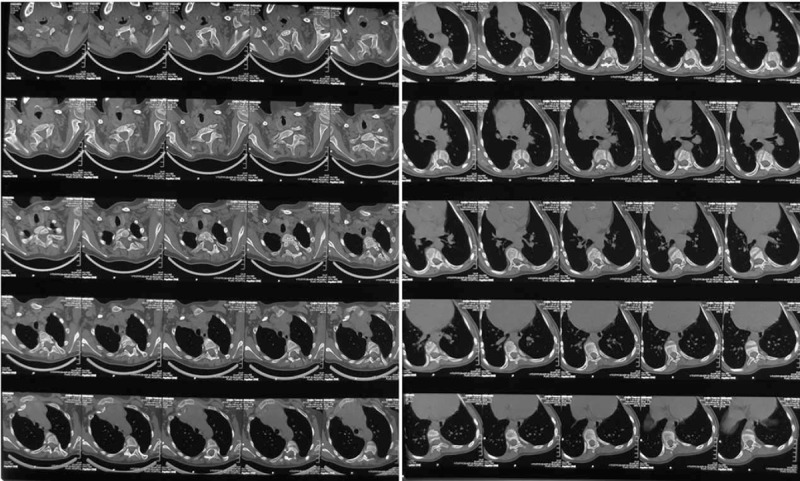
Computed tomography (CT) revealed a thoracic 5 and 6 vertebral body dysplasia.

**FIGURE 3:**
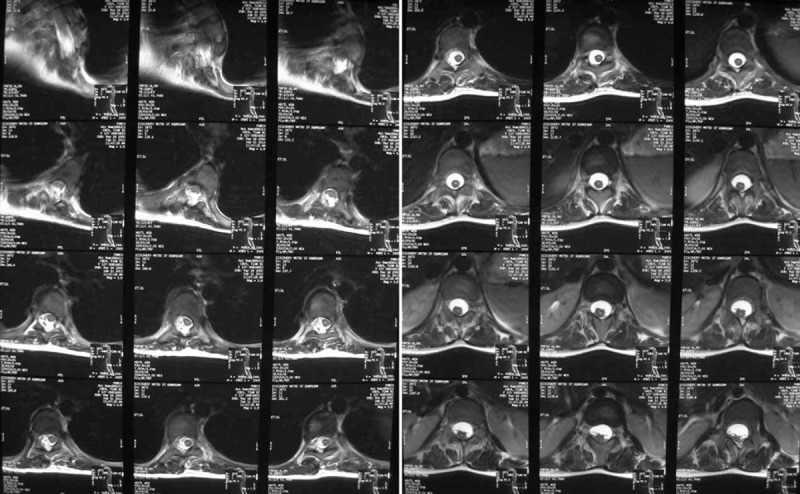
Magnetic resonance imaging (MRI) revealed syringomyelia.

Radiographs of the spine during the initial assessment in our clinic revealed a left upper thoracic scoliosis extending from T1 to T7 and measuring 74° and a right main thoracic scoliosis extending from T7 to L1 and measuring 60° (Figure 1). The Risser grade was I, indicating that the patient had a significant amount of remaining growth and this could result in further deterioration of her scoliosis. Computed tomography revealed a thoracic 5 and 6 vertebral body dysplasia and spinal stenosis during thoracic 1 to thoracic 5 (Figure 2). An ultrasound of the kidneys and the abdomen did not elicit pathological findings. An electrocardiogram examination showed right bundle-branch block. A cardiological examination showed mild pulmonary stenosis, right ventricular enlargement, and mild aortic valve thickening. A respiratory assessment showed restrictive ventilation dysfunction. She cannot have the magnetic resonance imaging (MRI) because of the steel wire which was used for total correction of Tetralogy of Fallot. Therefore, the steel wires were removed in the Department of Cardiac Surgery of our hospital. MRI revealed syringomyelia (Figure 3).

**FIGURE 4:**
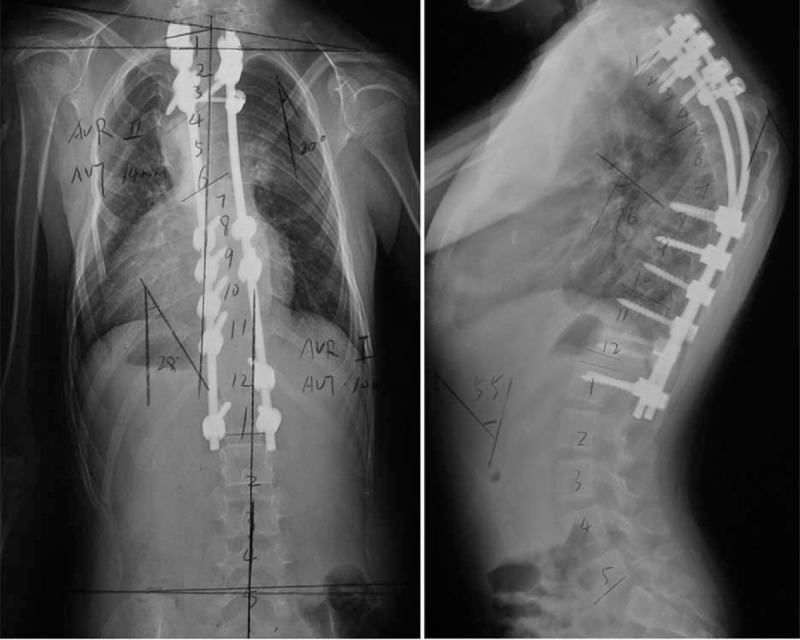
Standing anteroposterior and lateral radiographs of 4 days after operation.

**FIGURE 5:**
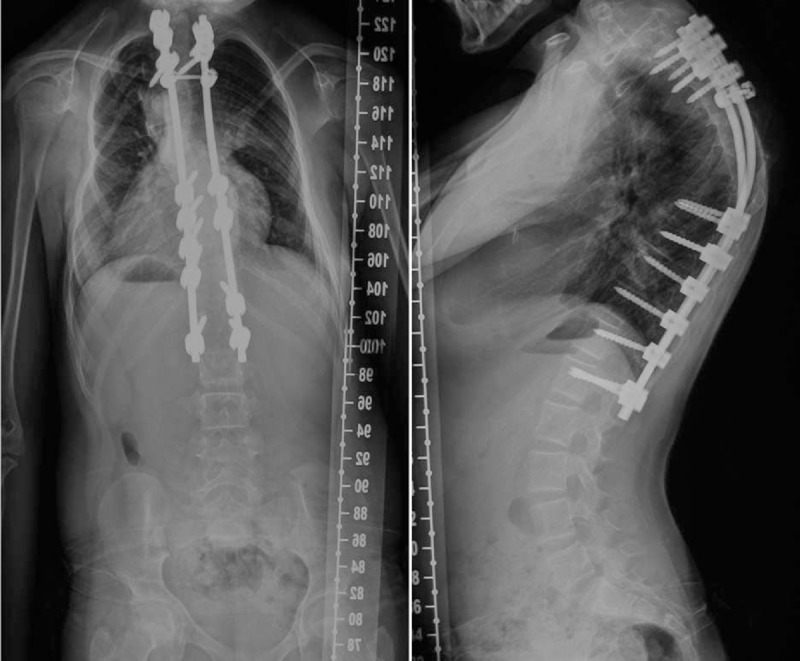
Standing anteroposterior and lateral radiographs of 12 months after operation.

In November 2013, a posterior correction and fusion at thoracic 1-lumbar 1 (T1-L1) levels were performed, using the Moss-SI spinal system (Johnson & Johnson, USA). The total operation time was about 5 hours. Total amount of blood loss was 600 mL. During the operation, the signal of this patient was normal using intraoperative spinal cord monitoring. Postoperatively, there was no sign of respiratory dysfunction. Postoperative plain X-ray film demonstrated a Cobb angles of upper thoracic correction from 74° to 20° (correction rate 73%) and Cobb angles of main thoracic correction from 60° to 28° (correction rate 37%) (Figure 4). Eight month following surgery, our patient developed a superficial dehiscence of the spinal wound, this was treated with resuturing and healed without further problems. There was no sign of a wound infection, and both the blood and wound cultures were negative. She was asymptomatic, well balanced in both the sagittal and coronal planes, with solid fusion at the next 6-month postoperative follow-up. Both the patient and families were satisfied with the results of surgery (Figure 5).

## DISCUSSION

The prevalence of SMS worldwide was estimated to be at 1:15,000 to 1:25,000 births.^11,12^ In addition to the major clinical findings mentioned previously, there are limited reports regarding the diagnosis and management of SMS with congenital scoliosis. In the present study, we reported the case of a 13-year-old SMS case with congenital scoliosis.

SMS is a rare genetic syndrome and caused by a deletion on chromosome 17 p11.2 or, more rarely, by a mutation of the *RAI1* gene located on this chromosome.^13^ SMS is characterized by distinctive facial appearance, a range of health problems and increased likelihood of behavioral problems including sleep disturbance, challenging behavior, stereotyped behaviors, impulsivity, and attention-seeking. SMS is a group of disorders marked by craniofacial, musculoskeletal, neurological, behavioral, and systemic abnormalities.^14,15^ There are various skeletal features in this syndrome, including persistent fetal finger pads, brachydactyly, cleft lip/palate, short stature, and polydactyly.^14^ However, there are some reports regarding the association with SMS and scoliosis. Spilsbury and Mohanty^14^ demonstrated that 7 of 22 SMS patients developed a scoliosis (30%). The curve types included 3 patients with right thoracic, 3 patients with left thoracolumbar, and 1 patient with double major. The curve sizes ranged from 18° to 113°. In addition, 1 patient also developed a thoracic lordosis. No radiological evidence of congenital vertebral anomalies was found. One patient demonstrated a rapidly progressive curve from 38° to 75° in 6 months. In all patients the scoliosis was stiff. Three of the 7 patients with a scoliosis were treated surgically due to progression of the deformity. Greenberg et al^16^ recorded the development of a mild-to-moderate scoliosis in 13 out of 20 patients (65%) of ≥4 years of age with SMS, and this was most commonly located in the mid-thoracic region but gave no information on the need for treatment. Tsirikos et al^8^ has reported a case of an 11-year-old British Caucasian girl with SMS who developed a severe, progressive thoracic, and lumbar scoliosis.

The exact etiology and mechanism of SMS with congenital scoliosis are still not known. The prevalence of scoliosis among patients with SMS was 30–65%, much higher than the incidence of scoliosis that among general population (2%).^14,16^ Retinoic acid (RA) signal pathway may play a crucial role in the development of SMS with congenital scoliosis. RA, the active form of vitamin A, is crucial in nuclear receptor signaling during development of several organs, including the hindbrain, spinal cord, heart, eye, skeleton, forelimb buds, lung, pancreas, and genitourinary tract.^17,18^ Moreover, recent studies showed that RA played a significant role in the development of somites in mouse, chick, or zebrafish embryos.^19–21^ In addition, our previous study also demonstrated that deficiency of vitamin A during pregnancy might result in congenital spinal deformities in the postnatal rats, which was caused by a defect in RA signaling pathway during somitogenesis.^22^ An interstitial 17p11.2 microdeletion is associated with SMS. In addition, the 17p11.2 region includes the *RAI1* gene (around 90% of cases) or *RAI1* gene point mutation (10% of cases of SMS).^1,23^ Therefore, the potential mechanisms in the pathogenesis of congenital scoliosis of SMS included RAI1 microdeletion and *RAI1* gene point mutation.

In conclusion, scoliosis is not uncommon among patients with SMS. Clinical symptom and special gene tests are needed to confirm the diagnosis. Surgeon and anesthesiologist must pay particular attention to the other organ abnormalities including cardiac, kidney, and brain problems. There is a potential association between congenital scoliosis and SMS. However, the exact mechanism how SMS induce scoliosis is unclear, the potential mechanisms in the pathogenesis of congenital scoliosis of SMS included RAI1 microdeletion and *RAI1* gene point mutation. Further studies with large patients sample size are needed to confirm the association between the 2 diseases. Animal models were needed to clarify its mechanisms.
